# The contribution of the English NHS Diabetic Eye Screening Programme to reductions in diabetes-related blindness, comparisons within Europe, and future challenges

**DOI:** 10.1007/s00592-021-01687-w

**Published:** 2021-04-08

**Authors:** Peter. H. Scanlon

**Affiliations:** 1 Gloucestershire Retinal Research Group, Office Above Oakley Ward, Cheltenham General Hospital, Sandford Road, Cheltenham, GL53 7AN UK; 2 Nuffield Department of Clinical Neuroscience, University of Oxford, Oxford, UK; 3 University of Gloucestershire, Cheltenham, UK

**Keywords:** Screening, Diabetic retinopathy, Vision impairment, Blindness

## Abstract

The aim of the English NHS Diabetic Eye Screening Programme (DESP) is to reduce the risk of sight loss amongst people with diabetes by the prompt identification and effective treatment if necessary of sight-threatening diabetic retinopathy, at the appropriate stage during the disease process, with a long-term aim of preventing blindness in people with diabetes.

For the year 2009–2010, diabetic retinopathy (DR) was no longer the leading cause of blindness in the working age group. There have been further reductions in DR certifications for WHO severe vision impairment and blindness from 1,334 (5.5% of all certifications) in 2009/2010 to 840 (3.5% of all certifications) in 2018/2019. NHS DESP is a major contributor to this further reduction, but one must also take into account improvements in glycaemic and blood pressure control, timely laser treatment and vitrectomy surgery, improved monitoring techniques for glycaemic control, and vascular endothelial growth factor inhibitor injections for control of diabetic macular oedema. The latter have had a particular impact since first introduced in the UK in 2013.

Current plans for NHS DESP include extension of screening intervals in low-risk groups and the introduction of optical coherence tomography as a second line of screening for those with screen positive maculopathy with two dimensional markers. Future challenges include the introduction of automated analysis for grading and new camera technologies.

## The English NHS Diabetic Eye Screening Programme

The NHS Diabetic Eye Screening Programme in England commenced in 2003 and achieved high population coverage and uptake by 2008. In 2017–2018, 2.70 million people with diabetes were offered screening [1] and 2.23 million screened (82.7%). This resulted in 8,782 urgent referrals and 54,893 routine referrals to ophthalmology departments. The screening method is two 45-degree field mydriatic digital photography per eye with screening and grading being undertaken by trained technicians or optometrists as previously described [2].

**Fig. 1:**
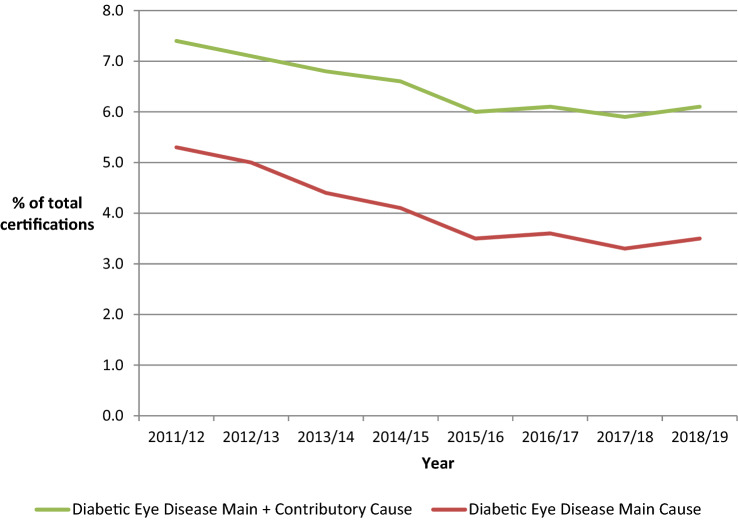
Percentage of new certifications of the combination of WHO severe vision impairment and blindness due to diabetic eye disease in England

In 2014, Liew [3] reported that, from an analysis of blindness certifications in the year 2009–2010, for the first time in at least five decades diabetic retinopathy/maculopathy was no longer the leading cause of certifiable blindness among working age adults in England. In 2013, an eye health indicator was incorporated into the Public Health Outcomes Framework [4] in England which resulted in ongoing annual reports being produced from certificates of vision impairment (CVIs) that are gathered and collated at Moorfields Eye Hospital, which have resulted in three further publications about blindness certifications in the UK [5–7]. In England, despite an overall increase in the numbers of certifications, the numbers that have diabetic eye disease as the main cause have shown a reduction from 1334 (5.5% of all certifications) in 2009/10 to 840 (3.5% of all certifications) in 2018/2019 (Table 1 and Fig. 1). The reduction has been principally in the age group 35 years and older from 1207 to 758, with an average of 51 per year in those aged 18–34 years which has shown little change over the same period.

**Table 1:** Number of new certifications of WHO of the combination of severe vision impairment and blindness due to diabetic eye disease in England

Year	2009/2010	2010/2011	2011/2012	2012/2013	2013/2014	2014/2015	2015/2016	2016/2017	2017/2018	2018/2019
Total number of Certificates of Visual Impairment (CVI)—all new certifications	22,687	22,501	23,616	22,647	22,911	23,017	22,973	23,453	22,844	24,284
Diabetic eye disease—single main cause	1261	1186	1263	1125	999	935	809	851	742	840
Diabetic eye disease—single main cause (% of Total)	*5.6*	*5.3*	*5.3*	*5.0*	*4.4*	*4.1*	*3.5*	*3.6*	*3.3*	*3.5*
Diabetic eye disease—single main cause—age 18–34 years	51	44	70	56	62	49	47	45	40	47
Diabetic eye disease—single main cause—age 35 years and above	1207	1140	1186	1058	932	883	758	805	695	789
Diabetic eye disease—single main and contributory cause	1703	1611	1756	1606	1569	1508	1383	1441	1343	1490
Diabetic eye disease—single main and contributory cause (% of total)	7.5	7.2	7.4	7.1	6.8	6.6	6.0	6.1	5.9	6.1

All ages (including age not stated and aged under 12)

The data provided by the Certifications Office (The Royal College of Ophthalmologists, c/o Certifications Office, Moorfields Eye Hospital) captured by the CVI are the copyright of the Department of Health and Social Care, and this work was made possible through a collaboration with the Royal College of Ophthalmologists. Any views expressed in this paper are those of the authors and not necessarily those of the Department of Health and Social Care

## Worldwide blindness due to diabetic retinopathy

Table 2 compares the different definitions that have been used in reporting blindness so that comparisons between studies can be more easily understood.

**Table 2:** Definitions of blindness and vision impairment

Registration	Category	Visual acuity in the better eye					
		Worse than			Equal to or better than		
	WHO criteria	6 m	20 Feet	LogMAR	6 m	20 Feet	LogMAR
	Mild vision impairment	6/12	20/40	0.3	6/18	20/60	0.48
	Moderate vision impairment	6/18	20/60	0.48	6/60	20/200	1.0
Legal blindness USA and many Western European countries Sight impaired Certification UK	WHO Severe vision impairment	6/60	20/200	1.0	3/60	20/400	1.3
Seriously sight impaired/legal blindness certification UK	WHO blindness	3/60	20/400	1.3			
	WHO Near vision impairment	N6 or M 0.8 at 40 cm					
	Categorisation by Central Visual Field:	Degrees					
	WHO Severe vision impairment	20					
	WHO blindness	10					

A 2020 publication [8] by a Vision Loss Expert Group of Collaborators reported that although diabetic retinopathy accounted for 0·86 million cases [0·59–1·23] of blindness in those aged 50 years and older in 2020, it was the smallest contributor to blindness in 2020 compared with under corrected refractive error, cataract, age-related macular degeneration, and glaucoma. However, it was the only cause of blindness that showed a global increase in age standardised prevalence between 1990 and 2020, which was of particular concern in younger, economically active age groups.

## Diabetes in Western Europe

In the Diabetes Atlas 2019 report [9], the age-adjusted comparative prevalence of diabetes in Western Europe was 6.3% expecting to rise to 7.3% in 2030.

In the UK, the National Diabetes Audit [10] from 2018–2019 recorded 7% of the population (3,537,385 people) with diabetes.

A recent publication [11] describing the implementation and 15-year follow-up of a population-based screening program in Andalusia in Southern Spain, which has a population of 8.4 million, reported that the prevalence of diabetes in Andalusia is higher (15.3%) than in the rest of Spain (12.5%).

## Blindness in Western Europe

In 2002, Kocur [12] reported that in people of working age in Europe, diabetic retinopathy was the most frequently reported causes of serious visual loss.

In 2012, Sivaprasad reported that minority ethnic communities with type 2 diabetes in the UK, in particular those of African/Afro-Caribbean’s and South Asian origin, are more prone to visual impairment including sight-threatening retinopathy and maculopathy [13], compared to white Europeans.

In 2018, Bourne [14] reported that the estimated number of people registered blind in Western Europe in 2015 was 1.16 (0.60–1.83) million and that 3.30 (0.47–7.60) % caused by DR suggesting that 38,280 people may be registered blind in Western Europe due to DR.

The 2020 publication [8] by a Vision Loss Expert Group of Collaborators report commented that there are surprisingly few data from high-income regions—only 19 studies included in the review reported cause-specific vision impairment in a high-income location, and all but three of these took place more than a decade ago.

Comparisons between studies that have been reported are made more difficult by the following:

### Populations studied

- Some studies are based on patients attending hospital clinics, and others are more population based.
- Other studies report on the numbers per 100,000 in the general population rather than on the numbers per 100,000 with diabetes.

### Incomplete reporting of data

In the UK, retrospective reviews of WHO severe vision impairment and blindness registrations have been made in several subpopulations and at a national level like in this article. Those registers that are held locally are more likely to be complete, but registration for an individual patient is still voluntary. There are more financial benefits for an individual who is registered WHO blind (severely sight impaired UK) than one who is registered as WHO severely visually impaired (sight impaired UK) which would suggest that the former may have more complete numbers than the latter. The national figures rely on data being sent to the certifications centre at Moorfields Eye Hospital which is very complete from some areas of the country, but there will be under reporting from other areas.

### Mortality of those with blindness due to DR

Only patients who were alive at follow-up may have been included in some studies even though it has been shown in the past that the mortality of those who have severe visual impairment or blindness is higher than those without [15].

### Patient consent

In studies requiring patient consent, those who have lost vision may decline to participate [15].

### Inclusion of blindness from other causes than diabetic retinopathy

Some studies [16, 17] included blindness from other causes than diabetic retinopathy in the population with diabetes. In 2003, the point prevalence [16] of legal blindness in Arhus County, Denmark, found was 0.6% for type 1 and 1.5% for type 2 diabetes patients. However, in type 1 diabetes patients, 66.2% of blind eyes were due to proliferative DR (PDR) and in type 2 diabetes, 21.9% was due to age-related macular degeneration, 18.5% diabetic maculopathy and 18% PDR.

### Summary of studies from Western Europe in populations with diabetes

Tables 3 and 4 include studies that could be converted to WHO definitions of severe visual impairment and blindness due to diabetic retinopathy and to numbers per 100,000 population with diabetes. Table 3 commences in 1993–1996 with two hospital-based studies showing reductions in blindness rates in Sweden [18, 19], followed by further reports reductions in blindness from Sweden [20] and Iceland [21] that they attributed to early detection of sight-threatening diabetic retinopathy by screening programmes. Studies by Nicolucci [22] in Italy, Cormack [23] in Scotland, and Kumar [24] in Leeds, England, provide background data of blindness levels in these areas at that time. In 2001, Trautner [25] reported reductions in blindness between 1990 and 1998 in people with diabetes in the area of Wurttemberg-Hohenzollern, Germany. In 2003 Arun [26] reported registration data from Newcastle, which was an area that had pioneered screening in the UK, and reported [27] figures from the working age population in 2009. Grausland [15] reported the 25-year cumulative crude incidence of blindness in type 1 diabetes was 7.5% (men, 8.0%; women, 6.8%; *P* = 0.61), corresponding to a mortality-adjusted cumulative incidence of blindness of 9.5% (95% CI, 7.1%–12.0%) and an overall incidence rate of blindness of 4.11 per 1000 person-years (95% CI, 3.03–5.59 per 1000 person-years). Further reports in Tables 3 and 4 include reductions in blindness related to diabetic retinopathy in Poland [28], Cambridge UK [29], Scotland [30], Ireland [31], Wales [32], Southern Germany [33], and Gloucestershire UK [34]. A report from Hungary [35] assessed WHO severe visual impairment and blindness levels using ‘Rapid Assessments of Avoidable Blindness’ (RAAB) in 105 clusters.

**Table 3:** Western European studies on blindness and severe visual impairment in populations with diabetes (1993–2006)

Year of publication	1993	1993	1996	1997	1997	1997	2000	2000	2001	2001	2001	2003	2006	2006	2006
Author	Agardh [18]	Agardh [18]	Henricsson [19]	Nicolucci [22]	Backlund [20]	Backlund [20]	Stefansson [21]	Stefansson [21]	Cormack [23]	Trautner [25]	Trautner [25]	Arun [26]	Kumar [24]	Bandurska [28]	Bandurska [28]
Location	Lund Sweden	Lund Sweden	Helsingborg Sweden	Italy	Stockholm Sweden	Stockholm Sweden	Iceland	Iceland	Fife Scotland UK	Wurttemberg-Hohenzollern Germany	Wurttemberg-Hohenzollern Germany	Newcastle UK	Leeds UK	Warmia Poland	Warmia Poland
Type of diabetes	Type 1	Type 2	Type 1 and 2	Type 1 and 2	Type 1 and 2	Type 1 and 2	Insulin depended (IDDM)	IDDM		Type 1 and 2	Type 1 and 2	Type 1 and 2	Type 1 and 2	Type 1 and 2	Type 1 and 2
Year of study	1990–1991	1990–1991	1990–1995	1993–1994	1990	1995	1980	1994	1999	1990	1998	1998–2000	2002	1989	2004
I or P*	I	I	I	P	I of referrals	I of referrals	P	P	P	I	I	I	I	I	I
Numbers within diabetes popul^n^	per 100,000	per 100,000	per 100,000	per 100,000	per 100,000	per 100,000	per 100,000	per 100,000	per 100,000	per 100,000	per 100,000	per 100,000	per 100,000	per 100,000	per 100,000
WHO SVI	500	600	100	2000			2400	500		72 (95% CI 61–82)	59 (95% CI 49–68)	56	81.7	102.4 (95% CI 65.7–139)	13.3 (95% CI 3.8–24.9)
WHO Blindness					0.63 (95% CI 0.47–0.83)	0.33 (95% CI 0.22–0.48)			210			35	33.7		

*I or P = Incidence or Prevalence

**Table 4:** Western European studies on blindness and severe visual impairment in populations with diabetes (2009–2019)

Year of publication	2009	2009	2009	2013	2013	2013	2016	2016	2017	2017	2018	2018	2018	2018	2019
Author	Arun[27]	Gordon-Bennett [29]	Grausland[15]	Hall[30]	Hall[30]	Hall[30]	Tracey [31]	Tracey [31]	Thomas [32]	Thomas [32]	Claessen[33]	Claessen[33]	Dale[34]	Dale[34]	Toth[35]
Location	Newcastle UK	Cambridge UK	Denmark(25 yr follow up)	Fife Scotland UK	Fife Scotland UK	Fife Scotland UK	Ireland	Ireland	Wales UK	Wales UK	Southern Germany	Southern Germany	Gloucestershire UK	Gloucestershire UK	Hungary 105 clusters RAAB
Type of diabetes	Type 1 and 2 15–64 yrs	Type 1 and 2	Type 1	Type 1 and 2	Type 1 and 2	Type 1 and 2	> 18 < 70 years	> 18 < 70 years	Type 1 and 2	Type 1 and 2	Type 1 and 2	Type 1 and 2	Type 1 and 2	Type 1 and 2	DM > 50 years
Year of study	2001- 2005	2004–05	2007–08	2000	2009	2009	2004	2013	2007–08	2014–15	2008	2012	2005- 08	2014- 17	2015
I or P*	I	I	I	I	I	P	I	I	I	I	I	I	I	I	P
Numbers within diabetes popul^n^	per 100,000	per 100,000	per 1000 person-years	per 100,000	per 100,000	per 100,000	per 100,000	per 100,000	per 100,000	per 100,000	per 100,000	per 100,000	per 100,000	per 100,000	per 100,000
WHO SVI	43	60	411 (95% CI, 303–559)				31.9 (95% CI 21.6–45.7)	14.9 (95% CI 8.2–25.1)	48.8	27.8	17.3 [95% CI 13.6–21.1)	8.9 [6.3–11.6] 16%	41.3 (95% CI 27.6–59.6)	10 (95% CI 5.1–17.9)	252
WHO Blindness	22	23		59.7	23.9	167			31.3	15.8			25.4 (95% CI 15.0 to 40.4)	2.0 (95% CI 0.3–6.7)	280

*I or P = Incidence or Prevalence

An Italian publication in 1994 reported [36] that diabetic retinopathy was the second most common cause of blindness (13.1%) in the province of Turin between 1967 and 1991 and the commonest cause of blindness in the age group 50–70. A further study [37] published in 2010 reported that diabetic retinopathy was the still the second most common cause of blindness (15%) in the province of Viterbo in 2002–2003.

In Finland, a National Register of Visual Impairment (VI) was established in 1982. A 2016 publication [38] reported on 4080 patients whose primary cause for vision impairment (VI) was DR using three 10-year cohorts (1982–1990, 1991–2000, 2001–2010). A significant change had occurred over the 10-year periods particularly in those diagnosed with proliferative diabetic retinopathy, characterised by an increasing age at the time of VI notification 39, 62, and 59, decreasing severity of VI with a lower proportion blind 42%, 22%, and 15% and higher age at death 54, 73, and 72 years. Although the register does not collect data on type of diabetes, despite the fact that there had been an increase over this time of insulin-treated T2DM, it is believed that there has been a genuine change in characteristics of those with T1DM. This is further supported by the observation that the proportion of VI related to DR in persons of working age had decreased from 15% in 1990 to 10% in 2010. The article also comments on an unchanged small number of blind patients with a median age of 29–31 yrs, similar to the findings in the UK of unchanged number of registrations reported in the 18–34 yrs age group, reflecting the problems in control of diabetes in some patients with diabetes in their late teens and twenties.

## Contributions to reductions of blindness in Western Europe

Landmark clinical trials and studies have shown the importance of the following factors in the development of sight-threatening diabetic retinopathy and diabetic macular oedema:

- 1. Glycaemic control [39–42]
- 2. Control of blood pressure [39, 40, 43, 44]

In addition, the following have reduced the incidence and prevalence of blindness:

- 3. Timely laser treatment for proliferative DR [45]
- 4. Timely laser treatment for clinically significant macular oedema [46]
- 5. Vitrectomy surgery [29]
- 6. Screening.

Early reports that screening and screening compliance were major contributors in preventing and/or reducing DR blindness came from Iceland [21], 47. This was reported on a larger scale when, 6 years after the introduction of the English Screening Programme, Liew [3] reported that, in the year 2009–2010, diabetic retinopathy/maculopathy was no longer the leading cause of certifiable blindness among working age adults in England. The 2019 World Report on Vision [48] produced by the World Health Organisation concluded that ‘this provides compelling evidence that systematic diabetic retinopathy screening, coupled with timely treatment of sight-threatening disease, can reduce vision impairment and blindness’.

Since 2010, further contributions to reductions in blindness are:

- 7. Vascular endothelial growth factor (VEGF) Inhibitor injections for diabetic macular oedema

VEGF inhibitor injections for diabetic macular oedema (DME) were available in England following approval by the National Institute of Health and Care Excellence (NICE)[49, 50] in 2013–2015, and in some other parts of Europe earlier than 2013 after they gained European Regulatory approval for diabetic macular oedema in 2010[51] and 2014[52]. A modelling study [53] in Japan calculated from 570,000 DME patients was included in a model over 5 years. Increased utilization of anti-VEGF agents resulted in 6,659 fewer cases of severe visual impairment (SVI; 26–35 ETDRS letters) or blindness (0–25 ETDRS letters) compared with the current care approach.

- 8. Intravitreal steroid treatments for DME that were available in England following approval [54, 55] by the National Institute of Health and Care Excellence in 2013–15.
- 9. More recent treatments to improve glycaemic control in type 1 [56] and type 2 diabetes [57]
- 10. Improved methods of monitoring [58] glycaemic control.

## Future developments and challenges for the English NHS Diabetic Eye Screening Programme

The current plans for the English NHS Diabetic Eye Screening Programme are.

- 1. Extension of screening intervals for low-risk groups based on their previous two screening results [59].

This is because we do not have easy access to other risk factor data and the most significant risk factor is what if any retinopathy was present on the most recent screening photographs [60].

- 2. The introduction of optical coherence tomography (OCT) in second-line digital surveillance clinics for those with screen positive diabetic maculopathy [61].

Our future challenges are:

- 1. The introduction of automated analysis for grading.

My own view is that we are most likely to introduce this at the DR/No DR level as they do in Scotland [62] to remove normal images from the grading queue in order to reduce the workload for graders in the English Screening Programme.

- 2. The assessment and introduction of new camera technologies for screening

If the new scanning confocal ophthalmoscopes are as good as is claimed [63] in the non-mydriatic format, there would be many advantages in introducing staged mydriasis into the English Screening Programme. At the present time, Scotland has to dilate 30% of their screening population [64] with higher numbers in older people with diabetes (62% ≥ 85yrs and 50% 75–84 yrs).

If we could find a camera that successfully photographs the area covered by the two 45 degree fields currently used by the English NHS DESP, the ungradable image rate without drops was < 10%, and this was shown to be cost-effective (i.e., was not prohibitively expensive), this would be very attractive to the programme. There are many factors that influence young people with diabetes in resisting attendance at screening, but removing the need for dilating eye drops may be one that could help attendance in this age group.

## Data Availability

The data provided by the Certifications Office (The Royal College of Ophthalmologists, c/o Certifications Office, Moorfields Eye Hospital) captured by the CVI are the copyright of the Department of Health and Social Care, and this work was made possible through a collaboration with the Royal College of Ophthalmologists. Any views expressed in this paper are those of the authors and not necessarily those of the Department of Health and Social Care.
